# MiR‐664‐3p suppresses osteoblast differentiation and impairs bone formation via targeting Smad4 and Osterix

**DOI:** 10.1111/jcmm.16451

**Published:** 2021-05-04

**Authors:** Yuexin Xu, Yucui Jin, Fangling Hong, Yunfei Ma, Jiashu Yang, Yuting Tang, Zhu Zhu, Jiahui Wu, Qianyi Bao, Lingyun Li, Bing Yao, Dong Li, Changyan Ma

**Affiliations:** ^1^ Jiangsu Key Laboratory of Xenotransplantation Nanjing Medical University Nanjing China; ^2^ Department of Medical Genetics Nanjing Medical University Nanjing China; ^3^ Department of Gynaecology and Obstetrics Northern Jiangsu People's Hospital Yangzhou China; ^4^ Jiangsu Key Laboratory of Oral Disease, Department of Oral Special Consultation, The Affiliated Stomatological Hospital of Nanjing Medical University Nanjing Medical University Nanjing China; ^5^ Department of Orthopedics, Jiangsu Province Hospital of Traditional Chinese Medicine Affiliated Hospital of Nanjing University of Traditional Chinese Medicine Nanjing China

**Keywords:** bone mineral density, miR‐664‐3p, osteogenic differentiation, ovariectomy, transgenic mice

## Abstract

Osteoporosis is a metabolic disorder characterized by low bone mass and deteriorated microarchitecture, with an increased risk of fracture. Some miRNAs have been confirmed as potential modulators of osteoblast differentiation to maintain bone mass. Our miRNA sequencing results showed that miR‐664‐3p was significantly down‐regulated during the osteogenic differentiation of the preosteoblast MC3T3‐E1 cells. However, whether miR‐664‐3p has an impact on bone homeostasis remains unknown. In this study, we identified overexpression of miR‐664‐3p inhibited the osteoblast activity and matrix mineralization in vitro. Osteoblastic miR‐664‐3p transgenic mice exhibited reduced bone mass due to suppressed osteoblast function. Target prediction analysis and experimental validation confirmed Smad4 and Osterix (Osx) are the direct targets of miR‐664‐3p. Furthermore, specific inhibition of miR‐664‐3p by subperiosteal injection with miR‐664‐3p antagomir protected against ovariectomy‐induced bone loss. In addition, miR‐664‐3p expression was markedly higher in the serum from patients with osteoporosis compared to that from normal subjects. Taken together, this study revealed that miR‐664‐3p suppressed osteogenesis and bone formation via targeting Smad4 and Osx. It also highlights the potential of miR‐664‐3p as a novel diagnostic and therapeutic target for osteoporotic patients.

## 1 INTRODUCTION

Bone homeostasis is a dynamic balance that includes bone formation by osteoblasts and bone resorption by osteoclasts.^1^ Once the orchestrated balance is destroyed, it causes various destructive bone diseases, including osteoporosis.^2^ Osteoporosis has become a global health problem, especially for postmenopausal women and ageing population.^3^

MiRNAs are a class of small non‐coding RNAs, around 22 nucleotides in length, which bind via incomplete or complete base pairing to specific sequences in the 3′‐untranslated region (3′UTR) or coding sequence (CDS) of mRNAs, inducing either translational repression or mRNA degradation.^4^, ^5^ MiRNAs have been reported to play diverse biological roles in regulating multiple physiological and pathological processes, including cellular differentiation, proliferation, glucose metabolism, cholesterol biosynthesis and cancer development.^6^, ^7^, ^8^, ^9^, ^10^ Increasing numbers of miRNAs have been implicated as regulators of different aspects of bone development. Certain miRNAs, such as miR‐139‐3p, miR‐34a and miR‐143, inhibit osteogenic differentiation,^11^, ^12^, ^13^ and conversely, miR‐200c, miR‐149 and miR‐196a promote osteogenic differentiation.^14^, ^15^, ^16^ Besides, several miRNAs have emerged as critical regulators of osteoclast biology.^17^ Although several studies have revealed that miRNAs regulate bone formation and bone resorption, thus contributing to bone homeostasis maintenance, most of these miRNAs have only been identified in vitro, and their functional roles in the pathophysiological mechanisms responsible for reduced bone formation in skeletal disorders remain to be established before they can be applied in a clinical setting. MiRNAs are also recognized as attractive therapeutic targets due to their size, known sequence and the fact that they can target multiple genes to subsequently alter cellular pathways and networks. In fact, a number of Phase I/II human clinical trials are underway towards testing the effects of miRNA mimics or antagomirs in vivo to treat specific diseases including cancers. For example, a locked‐nucleic‐acid‐modified anti‐miR‐122 drug Miravirsen was successfully tested in phase II clinical trials in anti‐HCV therapy.^18^ TargomiRs loaded with miR‐16 mimic were tested in phase I clinical trials in malignant pleural mesothelioma.^19^

Icariin (ICA), a prenylated flavonol glycoside isolated from the Epimedium pubescens, exerts beneficial effects on preventing postmenopausal bone loss and treating osteoporosis.^20^ Previously published data and our recent study indicated that ICA promoted osteogenic differentiation in vitro and alleviated osteoporosis in vivo.^21^, ^22^, ^23^ To identify novel miRNAs involved in bone formation, differential miRNA expression profiles between ICA‐treated and untreated MC3T3‐E1 cells were evaluated. MiR‐664‐3p was one of the most significantly down‐regulated miRNAs during osteogenic differentiation. Previous studies demonstrated that miR‐664‐3p was closely related to the occurrence and development of a variety of tumours.^24^, ^25^, ^26^ However, there have been no studies on the potential role of miR‐664‐3p in bone metabolism. In addition, two key regulators of osteogenesis, including *Smad4* and *Osterix (Osx)*, are the potential targets of miR‐664‐3p. On this basis, we chose miR‐664‐3p as a research object to explore its negative effect on osteoblast differentiation and bone formation. Our data reveal new regulatory mechanisms of bone metabolism and also suggest a potential diagnostic and therapeutic target for osteoporosis.

## 2 MATERIALS AND METHODS

### 2.1 Cell culture and osteoblast differentiation

Murine preosteoblast MC3T3‐E1 cells were purchased from Cell Bank of the Chinese Academy of Sciences (Shanghai, China) and cultured in α‐MEM (Gibco, Carlsbad, CA, USA). HEK 293T cells and murine embryonic mesenchymal stem cell line C3H10T1/2 were obtained from American Type Culture Collection (Manassas, VA, USA) and grown in DMEM (Gibco), respectively. All culture media were supplemented with 10% fetal bovine serum and 1% penicillin/streptomycin (Invitrogen, Carlsbad, CA, USA). Cells were incubated in a humidified atmosphere with 5% CO_2_ at 37°C. In vitro osteoblast differentiation of MC3T3‐E1 and C3H10T1/2 cells was carried out using osteogenic induction medium containing standard growth medium supplemented with 50 mg/L ascorbic acid, 10 mmol/L β‐glycerophosphate and 10 nmol/L dexamethasone (Sigma‐Aldrich, Louis, MO, USA).

### 2.2 Cell transfection

MiR‐664‐3p mimic (Mimic‐664) and its negative control (Mimic‐NC) were purchased from RiboBio (Guangzhou, China) and transfected into cells at a concentration of 100 nmol/L. For plasmid transfection, 2 μg DNA was used for each plasmid when cotransfected with miRNAs into cells in six‐well plates. All transfections were performed using Lipofectamine 2000 (Invitrogen), according to the manufacturer's instructions.

### 2.3 RNA isolation and quantitative real‐time reverse transcription PCR (qRT‐PCR)

Total RNA was isolated from cells using TRIzol reagent (Invitrogen) and reverse transcribed into cDNA using the PrimeScript RT Reagent Kit (Takara, Otsu, Japan). Real‐time PCR was performed using FastStart Universal SYBR Green Master (Roche, Indianapolis, IN, USA) on a LightCycler 96 Real‐Time PCR System. The amplification conditions were as follows: 95°C for 10 minutes, followed by 40 cycles of 95°C for 10 seconds and 60°C for 30 seconds. *β‐actin* was used as an endogenous control. The expression level of mature miR‐664‐3p was determined by stem‐loop qRT‐PCR as previously described.^27^ *U6* was used for normalization of miRNA qRT‐PCR data. The primers used are listed in Table S1 and S2.

### 2.4 Western blotting

Protein isolation and western blot analyses were performed as previously described.^28^ The primary antibodies used included OSX (1:4000; Abcam, Hong Kong, China), SMAD4 (1:1000; Proteintech, Wuhan, China) and β‐actin (1:10 000; Santa Cruz Biotechnology, Santa Cruz, CA, USA).

### 2.5 Dual‐luciferase reporter assay

The 3′UTR of mouse and human *Smad4*, the CDS region of mouse *Osx*, as well as the 3′UTR of human *Osx*, including the predicted miR‐664‐3p‒binding site, was amplified and cloned into pGL3‐Promoter vector (Promega, Madison, WI, USA), respectively. Mutations and deletions were generated by site‐directed mutagenesis, by replacing or deleting the ribonucleotides of the miR‐664‐3p complementary sequence. All constructions were confirmed by sequencing. The primers used are listed in Table S3. The wide‐type, deleted or mutated luciferase reporter constructs were cotransfected into HEK 293T cells with Mimic‐664 or Mimic‐NC using Lipofectamine 2000. Luciferase activities were measured using the Dual‐luciferase Reporter Assay Kit (Promega) following the manufacturer's protocol. Renilla luciferase was used as an internal control.

### 2.6 Enzyme‐linked immunosorbent assay (ELISA)

Blood was collected intraorbitally from mice, and sera were stored at −80°C. Five samples were obtained for each group. Serum osteocalcin (OCN) level was detected by ELISA (Amersham Pharmacia Biotech, Piscataway, NJ, USA).

### 2.7 High‐throughput sequencing

MC3T3‐E1 cells were treated with ICA (5 µmol/L, National Institute for the Control of Pharmaceutical Biological Products, Beijing, China) or dimethyl sulfoxide (DMSO) vehicle for 48 hours. Total RNA was isolated and miRNA sequencing was performed by Beijing Genomics Institute (Shenzhen, China). Differentially expressed miRNAs were identified by fold‐change screening.

### 2.8 ALP and alizarin red S (ARS) staining

Cells were cultured in osteogenic induction medium for 7 days and then stained using the BCIP/NBT Alkaline Phosphatase Color Development Kit (Beyotime Institute of Biotechnology, Shanghai, China) following the manufacturer's instructions. Cells were washed three times with phosphate‐buffered saline (PBS) and then microphotographed. The staining intensities were determined using ImageJ software (National Institutes of Health, Bethesda, NJ, USA). ARS staining was performed at day 21 post‐osteogenic induction. Cells were fixed with ethanol for 10 minutes and treated with 1% ARS solution (Sigma‐Aldrich) at 25°C. The stained calcified nodules were microphotographed. To quantitatively evaluate the mineralized nodules, the stain was dissolved in 1 mL 10% cetylpyridinium chloride (BioBomei, Hefei, China) for 1 hour and the absorbance at 570 nm was detected by spectrophotometric methods.

### 2.9 Generation of conditional miR‐664‐3p transgenic mice

CAG‒664‐3p‒Cas9 knock‐in mice were generated by the Model Animal Research Center of Nanjing University (Nanjing, China) using CRISPR/Cas9 technology. The single‐guide RNA (sgRNA): 5′‐CTGAGCCAACAGTGGTAGTA‐3′ with PAM sequence AGG was injected into the C57BL/6J mouse zygotes together with Cas9 and donor DNA. Founder 0 (F0) mice were mosaic, and mouse tail junction PCR was used to screen potential F0 mice with correct gene targeting, which were further bred with C57BL/6J mice. F1 mice were further screened by tail junction PCR to guarantee no random insertion as well as correct targeting. For mouse tail genotyping, three pairs of primers were used as shown in Table S4. The 5′ end and 3′ end primers produced a 1785 and 1543 bp band in knock‐in allele, respectively. The wild‐type primer generated a 480 bp band in wild‐type allele. The Tg (Col1a1‐Cre/ERT2) mutant mouse line (Stock # 016241) was purchased from Jackson Laboratory (Bar Harbor, Maine, USA). CAG‒664‐3p‒Cas9 knock‐in mice were bred with Col1a1‐Cre/ERT2 mice to generate osteoblastic miR‐664‐3p transgenic mice (cre, miR‐664‐3p^+/+^; cre, miR‐664‐3p^+/−^) or littermate control mice (miR‐664‐3p^+/+^) that carry only CAG‒664‐3p transgene. All mice (6‐week‐old) received tamoxifen injection on seven consecutive days and analysed 2 weeks later. Genotyping was performed using DNAs extracted from mice tails. miR‐664‐3p^+/+^ mice showed two bands: 1785 and 1543 bp; cre, miR‐664‐3p^+/−^ mice showed four bands: 1785, 1543, 480 and 118 bp; cre, miR‐664‐3p^+/+^ mice showed three bands: 1785, 1543 and 118 bp. All mice were raised in a thermostatic 12 hours/12 hours dark‐light cycle environment, with free access to food and water. All animal experiments were performed in accordance with the National Institutes of Health guidelines and were approved by the Ethics Committee on Animal Care of Nanjing Medical University (Nanjing, China).

### 2.10 Therapeutic inhibition of miR‐664 in OVX mice

All the female C57BL/6J mice used were maintained under standard animal housing conditions (12 hours light, 12 hours dark cycles and free access to food and water). The mice were ovariectomized or sham‐operated at 8 weeks of age. At 8 weeks after surgery, all mice were killed by carbon dioxide asphyxiation and the bilateral femurs were collected for micro‐computed tomography (μCT). For the therapeutic model, mice were ovariectomized or sham‐operated at 8 weeks of age and left untreated until they were 16 weeks old. The miR‐664‐3p antagomir (antagomir‐664) and its negative control (antagomir‐NC) obtained from RiboBio were dissolved in PBS and delivered by subperiosteal injection into the femur metaphysis at 1 nmol/mouse. Control groups were injected with same volume of PBS. All animal experiments were performed in accordance with the National Institutes of Health guidelines and were approved by the Ethics Committee on Animal Care of Nanjing Medical University (Nanjing, China).

### 2.11 Bone mineral density (BMD) and morphometry

The distal femurs were dissected free of soft tissue, fixed in 4% paraformaldehyde for 24 hours and scanned using μCT (SkyScan 1176; Bruker, Germany). Image acquisition was performed at 100 kV and 98 μA with a 0.98‐degree rotation between frames. The resolution of the μCT images was 18.2 μm. During scanning, the samples were enclosed in tightly fitting plastic wrap to prevent movement and dehydration. For visualization, the segmented data were imported and reconstructed as three‐dimensional renderings displayed in the 3D software that comes with the instrument. BMD (mg/cm^3^), bone volume/total volume (BV/TV), trabecular thickness (Tb.Th), trabecular bone number (Tb.N) and trabecular spacing (Tb.Sp) were measured directly using the μCT evaluation programme.

### 2.12 Collection of clinical samples

Peripheral blood samples were collected from women with fracture (n = 142) aged from 50 to 89 from two clinical settings (The First Affiliated Hospital of Nanjing University of Traditional Chinese Medicine, Northern Jiangsu People's Hospital). Subjects with diabetes mellitus, hyperprolactinaemia, OVX, liver cancer and malabsorption syndrome affecting bone metabolism were excluded from our study (exclusive criteria). Osteoporosis was defined as spine or hip BMD T‐score ≤ −2.5 standard deviation (SD) and the control was defined as spine or hip BMD T‐score ≥ −1.0 SD. All the clinical procedures were approved by the Ethics Committee of the First Affiliated Hospital of Nanjing University of Traditional Chinese Medicine and Northern Jiangsu People's Hospital. Written informed consent was obtained from all participants.

### 2.13 Statistical analyses

Adequate sample size was determined according to the previous studies that performed analogous experiments. Experimental data are presented as mean ± SD from at least three independent experiments. Two sets of data were analysed by two‐sided Student's *t* test. Two‐sided *P* values < 0.05 were considered statistically significant.

## 3 RESULTS

### 3.1 MiR‐664‐3p expression is down‐regulated during osteogenic differentiation

**FIGURE 1:**
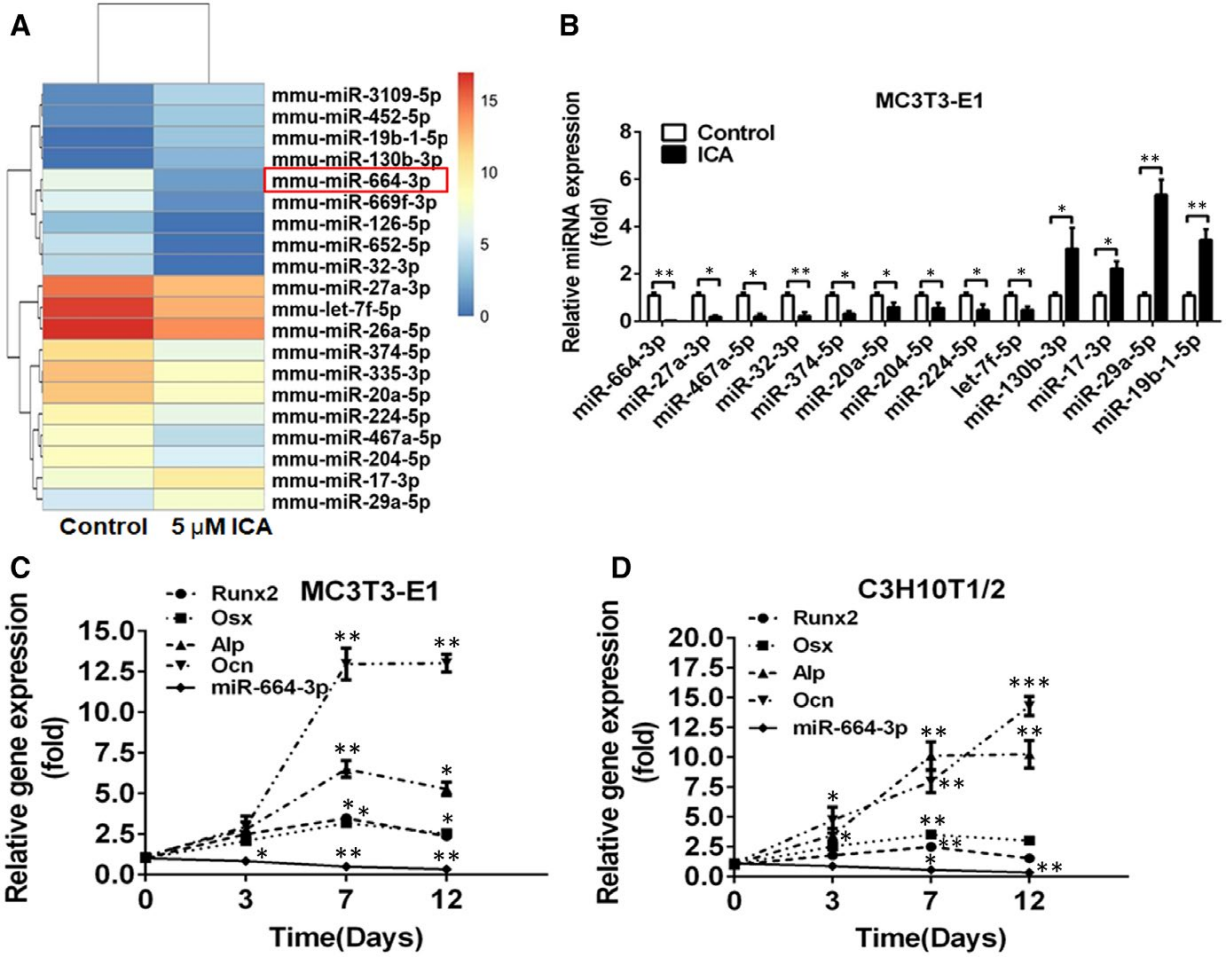
MiR‐664‐3p is down‐regulated during osteoblast differentiation. A, Alterations in miRNAs expression in ICA‐treated and untreated MC3T3‐E1 cells as examined by high‐throughput sequencing. Data displayed as red and blue represent elevated (high) and reduced expression (low), respectively. B, Stem‐loop qRT‐PCR for miR‐664‐3p, miR‐27a‐3p, miR‐467a‐5p, miR‐32‐3p, miR‐374‐5p, miR‐20a‐5p, miR‐204‐5p, miR‐224‐5p, let‐7f‐5p, miR‐130b‐3p, miR‐17‐3p, miR‐29a‐5p and miR‐19b‐1‐5p in ICA‐treated and untreated MC3T3‐E1 cells (normalized to the internal reference *U6*). C and D, The mRNA expressions of miR‐664‐3p, *Runx2*, *Osx*, *Alp*, and *Ocn* in MC3T3‐E1 (C) and C3H10T1/2 (D) cells incubated with osteogenic induction medium. Data are mean ± SD, n = 3. ^*^ *P* < 0.05, ^**^ *P* < 0.01 and ^***^ *P* < 0.001

Our previous study has proved that ICA, at 5 μmol/L concentration, exhibited the most prominent stimulatory effects on osteogenic differentiation of MC3T3‐E1 cells.^21^ To identify novel miRNAs that are associated with osteogenic differentiation, high‐throughput sequencing was performed to detect the differential miRNA expression after treatment with 5 μmol/L ICA in MC3T3‐E1 cells. The expression of 346 miRNAs was significantly altered (fold‐change > 2; *P* < 0.05), with 251 down‐regulated and 95 up‐regulated in ICA‐treated cells compared with the control cells (Figure 1A and Table S5). Among them, twenty miRNAs, with potential roles in osteoblast differentiation based on target and pathway prediction analysis, were selected for further validation by stem‐loop qRT‐PCR, and thirteen of them were validated, including miR‐20a‐5p, miR‐204‐5p, miR‐224‐5p, miR‐27a‐3p, miR‐32‐3p, miR‐374‐5p, miR‐467a‐5p, miR‐664‐3p, let‐7f‐5p, miR‐130b‐3p, miR‐17‐3p, miR‐19b‐1‐5p and miR‐29a‐5p (Figure 1B). Considering that miR‐664‐3p is at the top of the down‐regulated miRNAs during ICA‐induced osteoblast differentiation, and no report is available on the involvement of miR‐664‐3p in bone metabolism, we selected miR‐664‐3p for further study.

To confirm the decreased expression of miR‐664‐3p during osteogenesis, we examined the expression level of miR‐664‐3p in MC3T3‐E1 and C3H10T1/2 cells after incubation in osteogenic induction medium for 12 days. As expected, miR‐664‐3p was down‐regulated gradually in a time‐dependent manner in both cells. As expected, the early osteoblastogenic differentiation markers, such as *Runx2*, *Osx* and *Alp*, were remarkably increased at the early stages of culture (0‐7 days) and decreased at the later stages of culture (12 days); while the late osteoblastogenic differentiation marker *Ocn* was gradually increased during osteogenic differentiation (Figure 1C,D). In addition, we also found miR‐664‐3p was lowly expressed in the bone tissues derived from 8‐week‐old mice (Figure S1). These data indicated that miR‐664‐3p might have a negative effect on osteogenic differentiation and bone formation.

### 3.2 MiR‐664‐3p inhibits osteoblast activity and matrix mineralization

**FIGURE 2:**
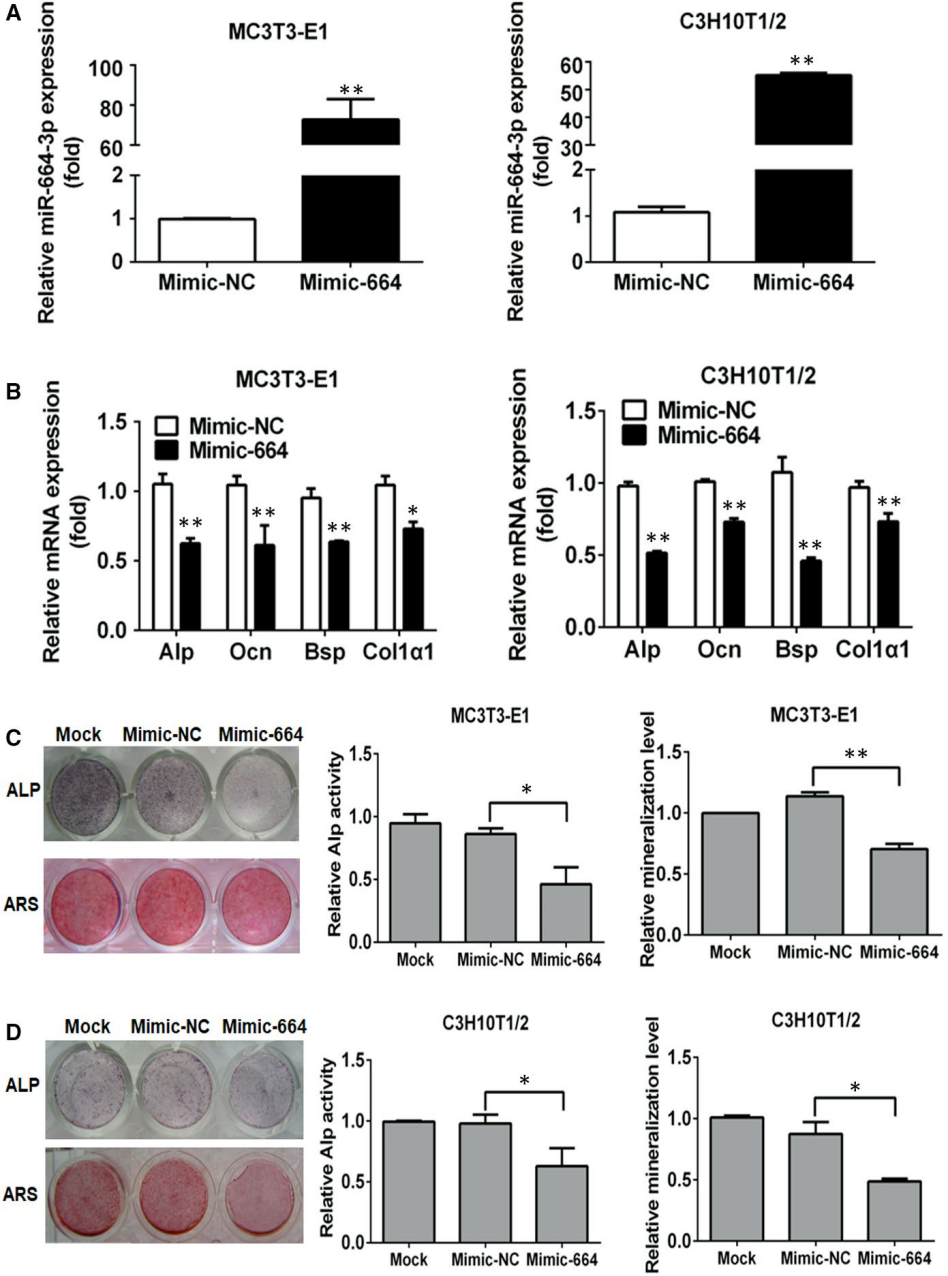
MiR‐664‐3p inhibits osteogenic differentiation in vitro. MC3T3‐E1 and C3H10T1/2 cells were transfected with 100 nmol/L Mimic‐664 or Mimic‐NC, and then incubated with osteogenic induction medium. A, The expression levels of miR‐664‐3p were determined by stem‐loop qRT‐PCR 48 h after transfection with Mimic‐664 or Mimic‐NC. B, The mRNA levels of osteogenic markers (*Alp*, *Ocn*, *Bsp* and *Col1a1*) were quantified by qRT‐PCR 48 h after transfection with Mimic‐664 or Mimic‐NC. C, Representative images of ALP and ARS staining of MC3T3‐E1 cells treated with Mimic‐664 or Mimic‐NC in osteogenic induction medium for 7 or 21 d, respectively. D, Representative images of ALP and ARS staining of C3H10T1/2 cells treated with Mimic‐664 or Mimic‐NC in osteogenic induction medium for 7 or 21 d, respectively. Data are mean ± SD, n = 3. ^*^ *P* < 0.05 and ^**^ *P* < 0.01

To explore the effects of miR‐664‐3p on osteoblast differentiation, MC3T3‐E1 and C3H10T1/2 cells were transfected with Mimic‐664, and then cultured in osteogenic induction medium. Intracellular miR‐664‐3p expression was markedly up‐regulated by Mimic‐664 transfection (Figure 2A). MiR‐664‐3p overexpression obviously decreased the mRNA levels of the key osteoblast markers, including *Alp*, *Ocn*, *Bsp* and *Col1a1* (Figure 2B). In addition, a significant reduction in ALP activity and matrix mineralization was observed in the Mimic‐664 group in both MC3T3‐E1 and C3H10T1/2 cells after osteogenic induction for 7 and 21 days, respectively (Figure 2C,D). Collectively, these data indicated that miR‐664‐3p was able to inhibit osteogenic differentiation and matrix mineralization in vitro.

### 3.3 MiR‐664‐3p inhibits in vivo bone formation

**FIGURE 3:**
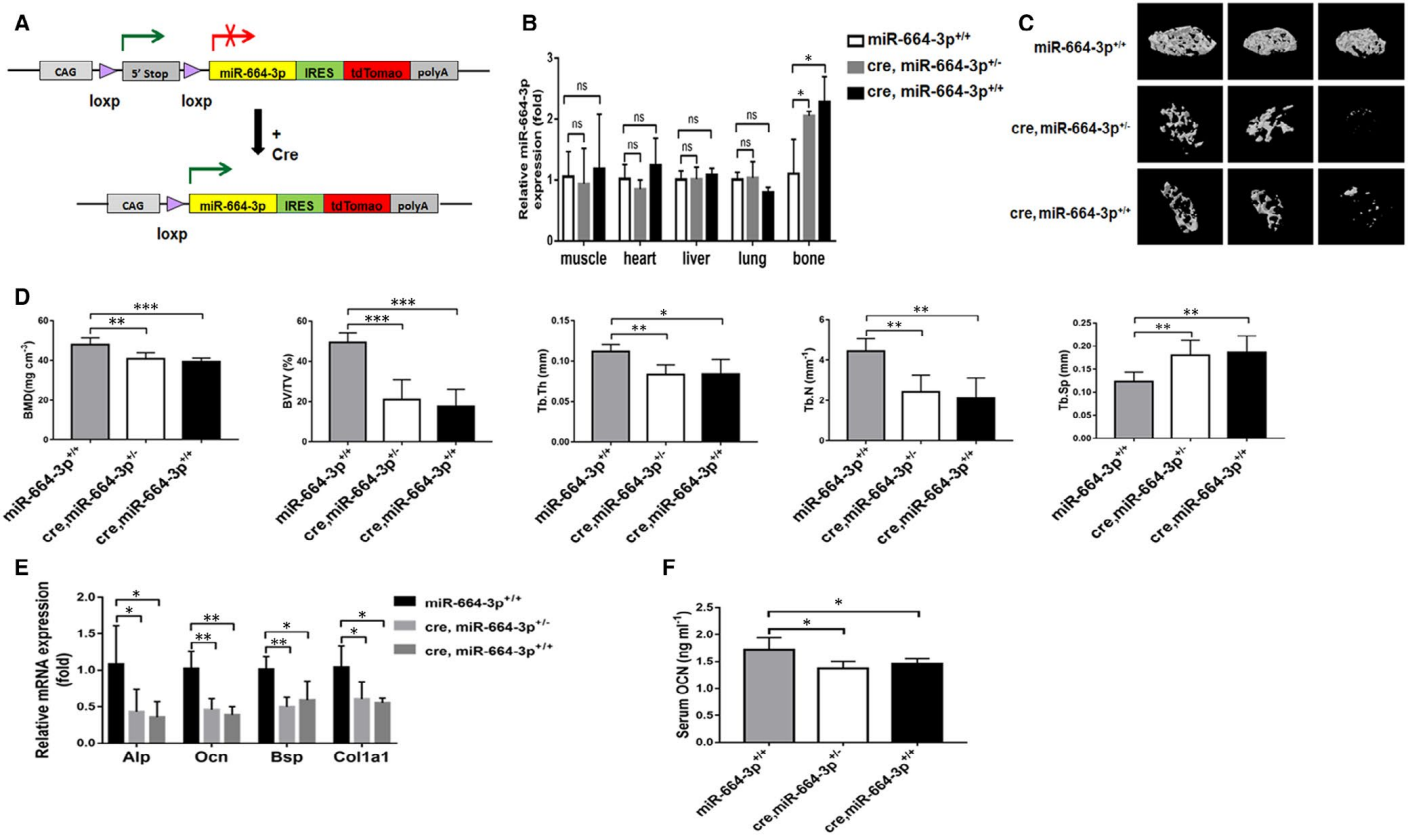
Characterization of bone phenotypes in osteoblast‐specific TG664 mice. A, Schematic representation of the generation of TG664 mice. B, qRT‐PCR analysis of miR‐664‐3p levels in bones and other tissues from two different TG664 mouse lines (TG664‐1: cre, miR‐664‐3p^+/−^ and TG664‐2: cre, miR‐664‐3p^+/+^) and control mice (miR‐664‐3p^+/+^). C, Representative μCT reconstructive images of the femoral metaphysis collected from indicated groups of mice. D, Bone morphometric analysis of trabecular bone of the distal femurs isolated from TG664 and control mice. BMD, bone mineral density; BV/TV, bone volume/tissue volume; Tb.Th, trabecular thickness; Tb.N, trabecular number; Tb.Sp, trabecular separation. E, qRT‐PCR analysis of *Alp*, *Ocn*, *Bsp* and *Col1a1* in bone tissues collected from TG664 and control mice at 9 weeks of age. F, ELISA assay to determine the serum concentrations of OCN in TG664 and control mice. Data are mean ± SD, n = 5 mice in each group. ^*^ *P* < 0.05, ^**^ *P* < 0.01 and ^***^ *P* < 0.001. ns, no significant difference

To further investigate the role of miR‐664‐3p in regulating bone formation in vivo, we generated osteoblastic miR‐664‐3p transgenic (TG664) mice by crossing CAG‒664‐3p‒Cas9 knock‐in mice with Col1a1‐Cre mice (Figure 3A and S2A). We selected two different TG664 mouse lines (TG664‐1: cre, miR‐664‐3p^+/−^ and TG664‐2: cre, miR‐664‐3p^+/+^) for further experiments. Their littermates (miR‐664‐3p^+/+^) were used as controls. The genotyping results are shown in Figure S2B. Compared to the control mice, both of TG664‐1 and TG664‐2 mouse lines showed higher miR‐664‐3p expression in bone tissues at 9 weeks of age (Figure 3B). μCT analysis showed that TG664 mice exhibited an osteoporotic bone phenotype accompanied by decreased trabecular bone volume, BMD, BV/TV, Tb.Th and Tb.N, and increased Tb.Sp, compared to their littermate controls (Figure 3C,D). To further explore whether the decreased BMD in TG664 mice was due to suppressed osteoblast function, we examined the expression levels of osteogenic marker genes. The mRNA expressions of Alp, Ocn, Bsp and Col1a1 were significantly lower in the whole femurs collected from TG664 mice compared with their littermate controls (Figure 3E). Consistently, the serum level of OCN, a marker for bone formation, was significantly lower in TG664 mice (Figure 3F).

### 3.4 MiR‐664‐3p directly targets Smad4 and Osx

**FIGURE 4:**
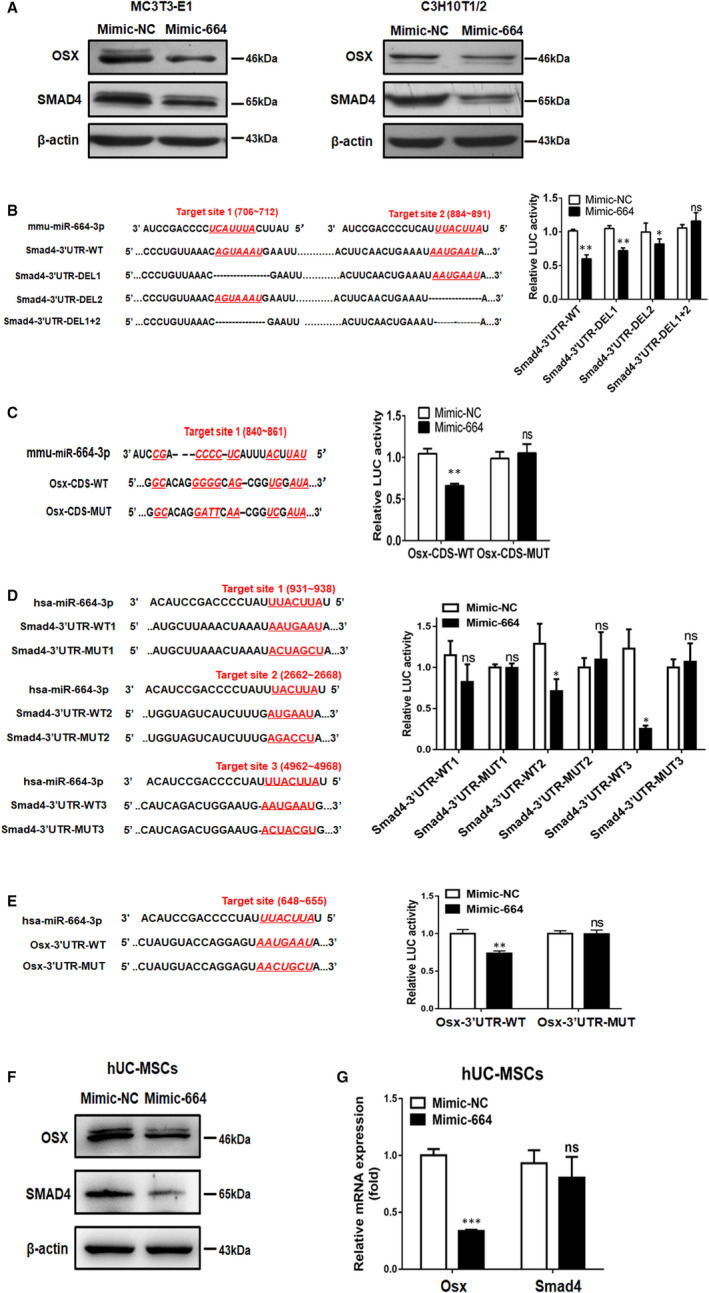
MiR‐664‐3p targets *Smad4* and *Osx*. A, Western blot analysis of SMAD4 and OSX protein levels in MC3T3‐E1 and C3H10T1/2 cells after treatment with Mimic‐664 or Mimic‐NC for 48 h. B, Schematic diagram of mmu‐miR‐664‐3p target sites in the *Smad4* 3′UTR and their deleted versions (left panel), and the luciferase activities of the WT and deleted *Smad4* reporters (*Smad4*‐3′UTR‐DEL1, *Smad4*‐3′UTR‐DEL2 and *Smad4*‐3′UTR‐DEL1+2) in HEK293T cells transfected with Mimic‐664 or Mimic‐NC for 48 h (right panel). C, Schematic diagram of mmu‐miR‐664‐3p target sites in the *Osx* CDS and their mutated versions (left panel), and the luciferase activities of the WT and mutated *Osx* reporter in HEK293T cells transfected with Mimic‐664 or Mimic‐NC for 48 h (right panel). D, Schematic diagram of hsa‐miR‐664‐3p target sites in the 3′UTR of human *Smad4* and their mutated versions (left panel), and the luciferase activities of the WT and mutated *Smad4* 3′UTR reporters in HEK293T cells transfected with Mimic‐664 or Mimic‐NC for 48 h (right panel). E, Schematic diagram of hsa‐miR‐664‐3p target site in the 3′UTR of human *Osx* and its mutated version (left panel), and the luciferase activities of the WT and mutated *Osx* 3′UTR reporter in HEK293T cells transfected with Mimic‐664 or Mimic‐NC for 48 h (right panel). F and G, Mimic‐664 or Mimic‐NC was transfected in hUC‐MSCs. The expression levels of SMAD4 and OSX were determined by western blotting (F) and qRT‐PCR (G). Data are mean ± SD, n = 3. ^*^ *P* < 0.05, ^**^ *P* < 0.01 and ^***^ *P* < 0.001. WT, wide‐type. DEL, deleted. MUT, mutated. ns, no significant difference

To elucidate the molecular mechanisms underlying miR‐664‐3p mediated inhibition of osteoblast differentiation and bone formation, we searched for potential targets of miR‐664‐3p using the miRNA target prediction algorithms (miRDB, TargetScan and RNA22, Table S6). Among the candidate target genes, we selected *Smad4* and *Osx* for further study because they are involved in the regulation of osteogenic differentiation.^29^, ^30^ We firstly examined *Smad4* and *Osx* expression in response to Mimic‐664 transfection in both MC3T3‐E1 and C3H10T1/2 cells. MiR‐664‐3p overexpression significantly down‐regulated endogenous SMAD4 and OSX protein expression (Figure 4A). By contrast, no change in *Smad4* and *Osx* mRNA levels was noted (Figure S3). Moreover, the intraosseous amounts of SMAD4 and OSX protein were much lower in the bone tissues collected from TG664 mice than control mice (Figure S4), suggesting that miR‐664‐3p regulated *Smad4* and *Osx* gene expression on the basis of translational repression rather than mRNA degradation.

To determine whether miR‐664‐3p targets *Smad4* directly, we constructed luciferase reporters containing either a wide‐type (WT) or deletion recognition sequence for miR‐664‐3p in the 3′UTR region of *Smad4*. Luciferase reporter assay demonstrated that Mimic‐664 treatment decreased the luciferase activity of *Smad4*‐3′UTR‐WT. Deletion of either miR‐664‐3p putative binding sites only partially abolished miR‐664‐3p‐mediated repression of luciferase activity, whereas deletion of both miR‐664‐3p binding sites completely abolished miR‐664‐3p‐mediated repression of luciferase activity (Figure 4B). These data indicated that miR‐664‐3p repressed *Smad4* expression by directly recognizing the two seed regions in the 3′UTR of *Smad4* mRNA. To determine whether miR‐664‐3p targeted *Osx* directly, we constructed luciferase reporters containing either a WT or mutant (MUT) recognition sequence for miR‐664‐3p in the CDS region of *Osx*. Luciferase reporter assay demonstrated that Mimic‐664 treatment inhibited the luciferase activity of *Osx*‐CDS‐WT, but not that of *Osx*‐CDS‐MUT, indicating that miR‐664‐3p targeted *Osx* through directly binding to the seed region in the CDS region of *Osx* mRNA (Figure 4C).

Since miR‐664‐3p is evolutionarily conserved among several species (Figure S5), we also investigated whether miR‐664‐3p targets *Smad4* and *Osx* in humans. Computational analysis revealed that the 3′UTR of human *Smad4* mRNA has three putative binding sites for hsa‐miR‐664‐3p and the 3′UTR of human *Osx* mRNA has one putative binding site for hsa‐miR‐664‐3p (Figure 4D,E). Luciferase reporter assay demonstrated that Mimic‐664 treatment significantly blocked the luciferase activities of human *Smad4*‐3′UTR‐WT2 and *Smad4*‐3′UTR‐WT3, but did not affect those of their respective mutated versions. However, Mimic‐664 treatment exerted no effects on *Smad4*‐3′UTR‐WT1 and its mutated version. These data implied that miR‐664‐3p targeted *Smad4* through directly binding to the second and third putative binding sites in the 3′UTR of human *Smad4* mRNA (Figure 4D). In addition, Mimic‐664 treatment down‐regulated the luciferase activity of the 3′UTR of *Osx* reporter, whereas mutations in the seed region of miR‐664‐3p in the 3′UTR of *Osx* abolished the regulatory effect of miR‐664‐3p on the 3′UTR of *Osx* (Figure 4E). We also examined the mRNA and protein levels of *Smad4* and *Osx* in response to Mimic‐664 transfection in human umbilicalcord mesenchymal stem cells (hUC‐MSCs). MiR‐664‐3p overexpression significantly decreased SMAD4 and OSX protein levels (Figure 4F), and decreased the mRNA level of *Osx*, but not *Smad4* (Figure 4G). Altogether, these results demonstrated that *Smad4* and *Osx* are the targets of miR‐664‐3p in both mouse and human.

### 3.5 Inhibition of osteogenic differentiation by miR‐664‐3p is partially mediated by Smad4 and Osx

**FIGURE 5:**
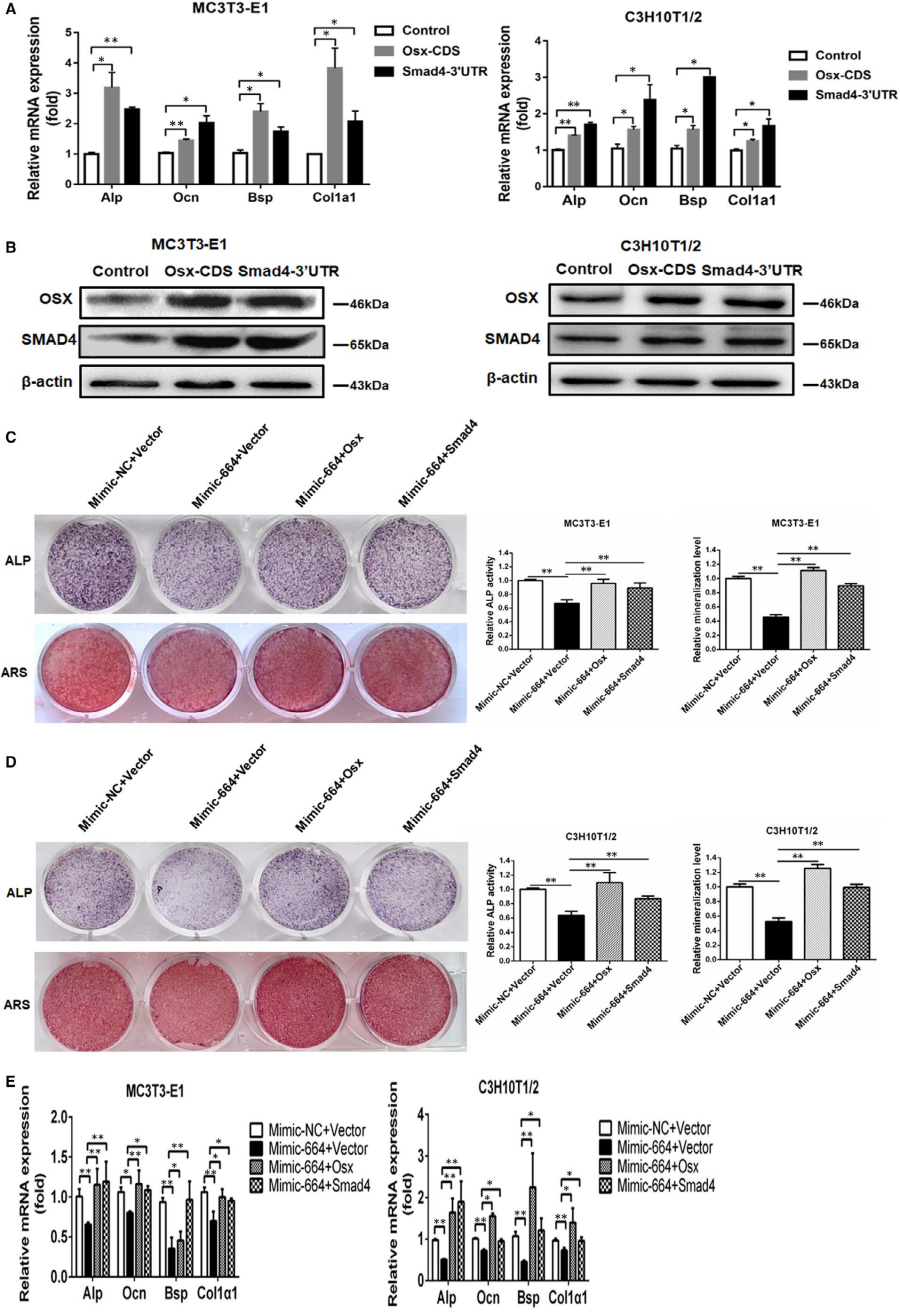
Inhibition of osteogenic differentiation by miR‐664‐3p partially depends on *Smad4* and *Osx*. A and B, WT *Smad4* 3′UTR or *Osx* CDS was transfected into MC3T3‐E1 and C3H10T1/2 cells, and then cultured in osteogenic induction medium for 48 h, respectively. The mRNA expressions of *Alp*, *Ocn*, *Bsp* and *Col1a1* were determined by qRT‐PCR (A). SMAD4 and OSX proteins were quantified by western blotting (B). C and D, Representative images of ALP and ARS staining in MC3T3‐E1 (C) and C3H10T1/2 (D) cells transfected with Mimic‐664 and *Smad4* or *Osx* overexpression plasmid in osteogenic induction medium for 7 or 21 d, respectively. E, qRT‐PCR analysis of *Alp*, *Ocn, Bsp* and *Col1a1* mRNA levels in MC3T3‐E1 and C3H10T1/2 cells transfected with Mimic‐664 and *Smad4* or *Osx* overexpression plasmid in osteogenic induction medium for 48h. Data are mean ± SD, n = 3. ^*^ *P* < 0.05 and ^**^ *P* < 0.01

To determine whether miR‐664 functionally targets *Smad4* and *Osx* in regulating osteoblast activity, we used the WT *Smad4* 3′UTR and *Osx* CDS to block the binding of endogenous miR‐664‐3p to *Smad4* and *Osx*, respectively. Results showed that *Alp*, *Ocn*, *Bsp*, and *Col1a1* mRNA levels were markedly higher in MC3T3‐E1 and C3H10T1/2 cells transfected with *Smad4* 3′UTR or *Osx* CDS compared to cells transfected with empty vector (Figure 5A). SMAD4 and OSX protein levels were up‐regulated by transfection with *Smad4* 3′UTR or *Osx* CDS (Figure 5B). Furthermore, Mimic‐664 was cotransfected with *Smad4* or *Osx* overexpression plasmid into MC3T3‐E1 and C3H10T1/2 cells to test whether *Smad4* and *Osx* are critical downstream targets responsible for the miR‐664‐3p function in osteoblastogenesis. The ALP and ARS staining results showed that ALP activity and calcification were suppressed by Mimic‐664, but the inhibitory effects could be reversed by *Smad4* or *Osx* overexpression on day 7 and 21, respectively (Figure 5C,D). Consistent with the results above, mRNA expressions of *Alp*, *Ocn*, *Bsp* and *Col1a1* decreased in Mimic‐664 group, but they were close to normal level (Mimic‐NC) after overexpressing *Smad4* or *Osx* (Figure 5E).

### 3.6 Inhibition of miR‐664‐3p partially counteracts the decreased bone phenotype in OVX‐induced osteoporotic mice

**FIGURE 6:**
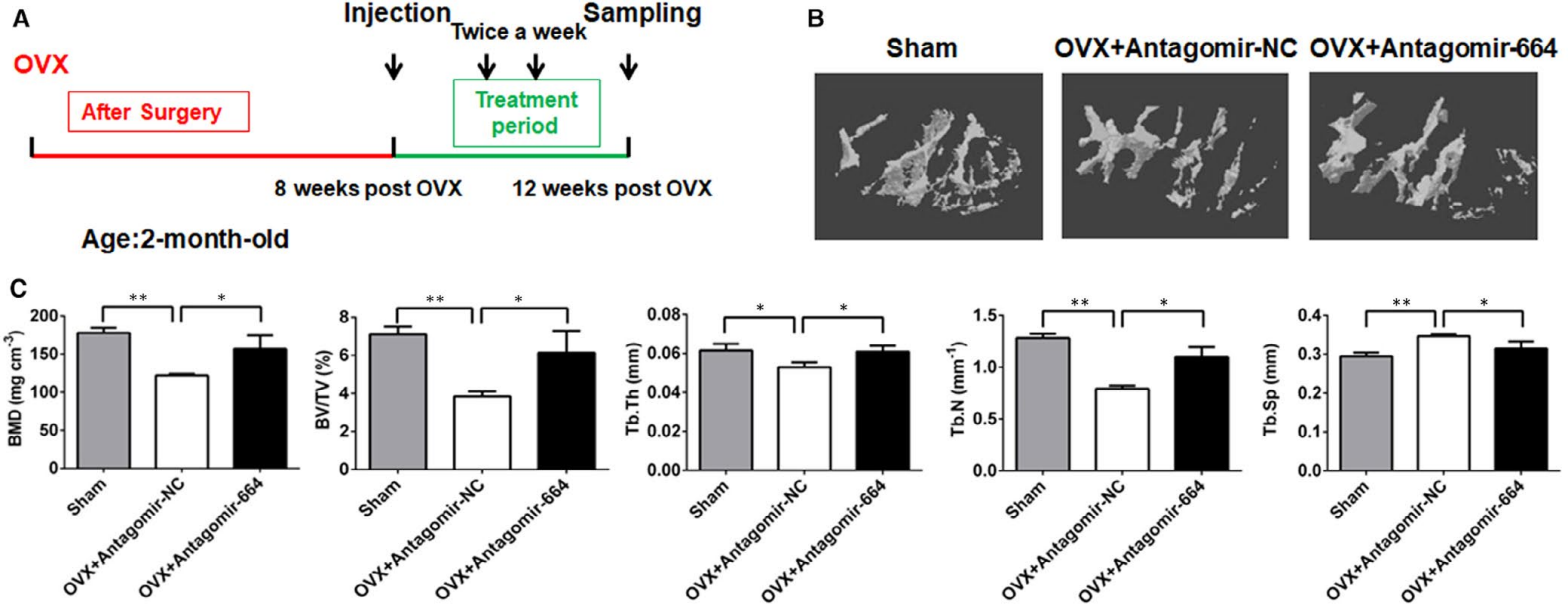
Therapeutic inhibition of miR‐664‐3p counteracts decreased bone phenotype in OVX‐induced osteoporotic mice. A, A schematic diagram illustrating the experimental design for the timeline of subperiosteal injection of antisense oligonucleotides specific to miR‐664‐3p and its negative control. B, Representative μCT reconstructive images of the femoral metaphysis collected from indicated groups of mice. C, Bone morphometric analysis of trabecular bone of the distal femurs isolated from each group. BMD, bone mineral density; BV/TV, bone volume/tissue volume; Tb.Th, trabecular thickness; Tb.N, trabecular number; Tb.Sp, trabecular separation. Data are mean ± SD, n = 5 mice in each group. ^*^ *P* < 0.05 and ^**^ *P* < 0.01

Numerous clinical studies have shown that the miRNAs associated with bone metabolism are implicated in osteoporosis.^31^, ^32^, ^33^ We chose an oestrogen‐deficient ovariectomized model to mimic postmenopausal osteoporosis.^34^ OVX or sham operation was performed on 8‐week‐old female mice. Eight weeks after surgery, μCT analysis of the femurs revealed significant trabecular bone loss, decrease of BMD, BV/TV, Tb.Th and Tb.N, and increase of Tb.sp in the OVX group compared with the sham group (Figure S6A,B). To investigate the therapeutic effects of inhibition of miR‐664‐3p on OVX‐induced osteoporosis, antagomir‐664 was injected into the femur metaphysis of oestrogen‐depleted mice 8 weeks after OVX (OVX+Antagomir‐664) (Figure 6A). μCT analysis revealed that antagomir‐664 treatment reversed OVX‐induced trabecular bone loss (Figure 6B). Additionally, μCT data quantification revealed that OVX+Antagomir‐664 mice exhibited increased BMD, BV/TV, Tb.N and Tb.Th, and decreased Tb.Sp at the distal femoral metaphysis compared to OVX+Antagomir‐NC mice (Figure 6C). These results indicated that miR‐664‐3p silencing was able to reverse the osteoporotic phenotype in OVX mice.

### 3.7 MiR‐664‐3p is up‐regulated in osteoporotic individuals

**FIGURE 7:**
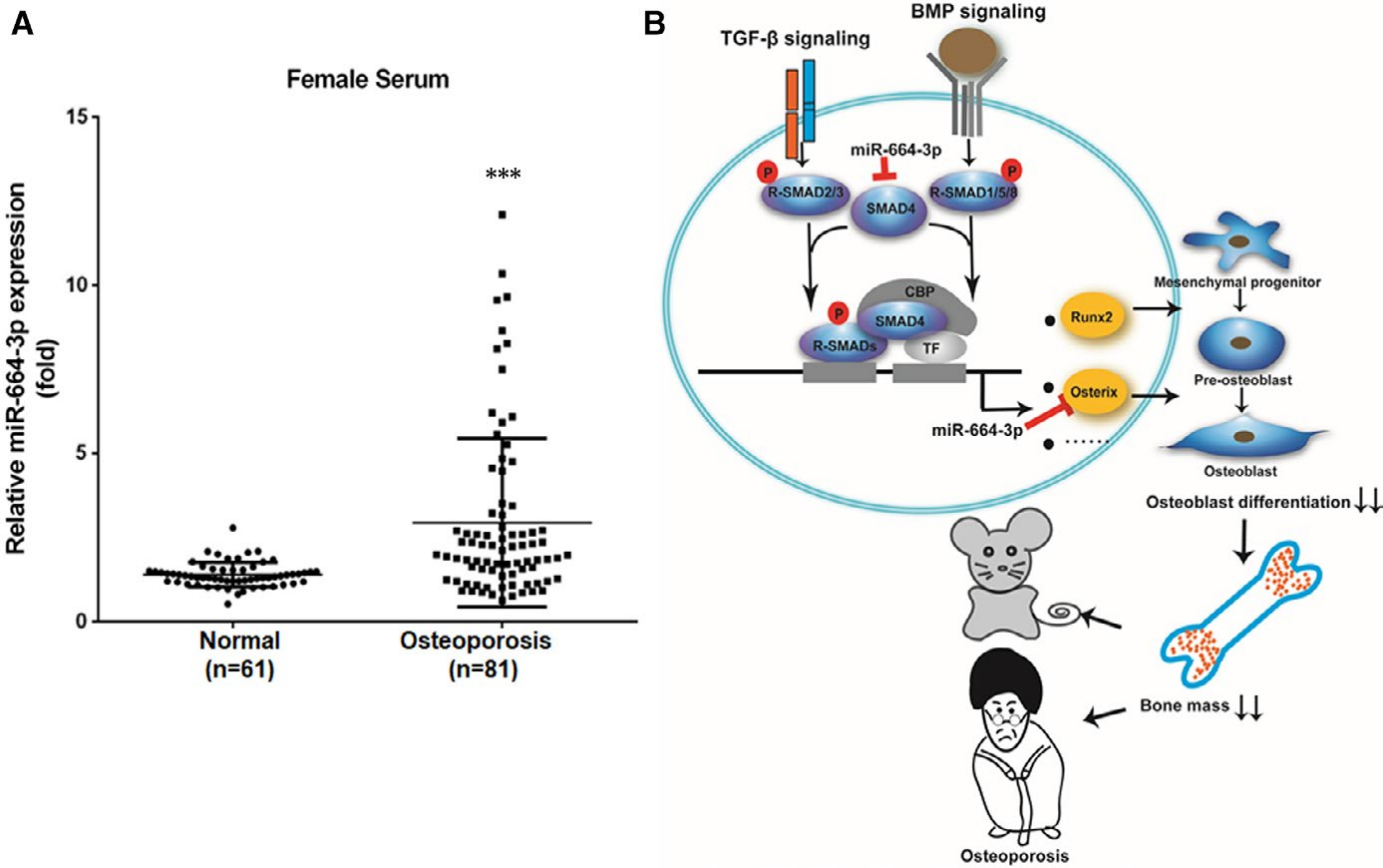
MiR‐664‐3p is up‐regulated in the serum of patients with osteoporosis. A, qRT‐PCR analysis of miR‐664‐3p levels in serum samples from female patients with osteoporosis and healthy controls. B, Schematic diagram representing the role of miR‐664‐3p in osteoblast differentiation and bone formation. ^***^ *P* < 0.001

Given that miR‐664‐3p silencing prevents OVX‐induced osteoporosis, we further evaluated the expression level of miR‐664‐3p in serum collected from osteoporotic patients and healthy donors. The serum samples were collected from 81 osteoporotic patients (T‐score ≤ −2.5) and 61 non‐osteoporotic patients (T‐score ≥ −1). The stem‐loop qRT‐PCR results showed that miR‐664‐3p was highly enriched in the serum of osteoporotic patients as compared with healthy donors (Figure 7A).

## 4 DISCUSSION

In this study, we identified the differentially expressed miRNAs during the osteogenic differentiation of MC3T3‐E1 cells using high‐throughput sequencing. The expression of 346 miRNAs was significantly altered. Among them, miR‐664‐3p was one of the most significantly down‐regulated miRNAs during the osteogenic differentiation of MC3T3‐E1 cells. MiR‐664‐3p has been reported to be deregulated in various human tumour types, including hepatocellular carcinoma, cutaneous squamous cell carcinoma and breast carcinoma.^24^, ^25^, ^26^ The expression level of miR‐664‐3p was positively correlated with longer lifespan in mouse and human populations.^35^, ^36^ However, the role of miR‐664‐3p in osteogenic differentiation has not been previously reported. Here, we identified a novel role of miR‐664‐3p in the inhibition of osteogenesis, that is, the forced expression of miR‐664‐3p significantly decreased ALP activity, calcified nodule formation and expression of bone‐related genes, including *Alp*, *Ocn*, *Bsp* and *Col1a1*. These data suggest that miR‐664‐3p is a negative regulator of osteoblast differentiation.

Smad4, a crucial transducer of TGFβ and BMP pathways, plays an important role in transducing TGFβ and BMP signals by forming intracellular signalling complexes with phosphorylated receptor‐regulated Smads (Smad 1, 2, 3, 5 and 8). The complexes then translocate into the nucleus and subsequently regulate the transcription of target genes, such as *Runx2* and *Osx*.^37^, ^38^ Several studies have demonstrated that miRNAs modulated the TGFβ and BMP pathway in the osteoblast lineage by targeting signalling ligands,^39^, ^40^ signalling receptors^41^, ^42^ and signal transducing Smads.^43^ In our present study, not only *Smad4*, but also another TGFβ and BMP signalling component *Osx*, are direct targets of miR‐664‐3p. Moreover, miR‐664‐3p‒mediated inhibition of osteoblast differentiation was remarkably attenuated by *Smad4* or *Osx* overexpression, as demonstrated by increased expression of *Alp*, *Ocn*, *Bsp* and *Col1a1*, and induction of ALP activity and calcified nodule formation, suggesting that miR‐664‐3p negatively modulates osteoblast differentiation in partially by inhibiting TGFβ and BMP pathways.

To determine whether miR‐664‐3p is a physiologically relevant regulator of bone formation, we also generated osteoblastic miR‐664‐3p transgenic mice. The TG664 mice exhibited osteoporotic bone phenotypes and lower levels of SMAD4 and OSX proteins compared to control mice. In addition, the expression levels of *Alp*, *Ocn*, *Bsp* and *Col1a1* were significantly lower in the femurs of TG664 mice; and similarly, serum OCN level was obviously decreased in TG664 mice. These data suggest that miR‐664‐3p impaired bone formation, which was partially due to suppressed osteoblast function. Furthermore, we constructed an ovariectomized mouse model to assess the efficacy of therapeutic targeting of miR‐664‐3p to prevent osteoporosis. μCT revealed an obvious decrease in bone mass in the OVX group, indicating successful establishment of the model. Treatment with antagomir‐664 markedly counteracted the decreased bone phenotype in OVX‐induced osteoporotic mice. These data indicate that inhibition of miR‐664‐3p might be a new strategy to treat age‐related bone loss and senile osteoporosis.

Most clinical investigations have been performed using bone samples from patients; however, it is not feasible for non‐invasive, early diagnosis of osteoporosis. In this study, we observed high enrichment of miR‐664‐3p in the serum from patients with osteoporosis, suggesting that serum miR‐664‐3p alone or in combined with other biomarkers may be used for osteoporosis diagnosis. However, to better explore the diagnostic roles of miR‐664‐3p, the expression pattern of miR‐664‐3p during progression from osteopenia to osteoporosis should be monitored in clinical trials in future.

In summary, we demonstrated that miR‐664‐3p suppressed osteoblast differentiation and impaired bone formation partially via regulation of *Smad4* and *Osx* expression. Silencing of miR‐664‐3p reversed OVX‐induced osteoporosis in vivo (Figure 7B). Moreover, we observed a significant enrichment of miR‐664‐3p in the serum of osteoporotic patients. Hence, this study is the first to reveal the role of miR‐664‐3p in osteogenic differentiation and bone formation, and also highlights the significance of miR‐664‐3p in the diagnosis and therapy of osteoporosis.

## CONFLICT OF INTEREST

The authors report no conflict of interest.

## AUTHOR CONTRIBUTIONS

**Yuexin Xu:** Formal analysis (lead); Investigation (lead); Validation (lead). **Yucui Jin:** Formal analysis (equal); Funding acquisition (supporting); Investigation (lead); Validation (lead); Writing‐original draft (lead). **Fangling Hong:** Formal analysis (equal); Investigation (equal); Validation (equal). **Yunfei Ma:** Formal analysis (supporting); Investigation (supporting). **Jiashu Yang:** Formal analysis (supporting); Investigation (supporting). **Yuting Tang:** Formal analysis (supporting); Investigation (supporting). **Zhu Zhu:** Resources (lead). **Jiahui Wu:** Formal analysis (supporting); Investigation (supporting). **Qianyi Bao:** Investigation (supporting); Methodology (supporting). **Lingyun Li:** Writing‐review & editing (supporting). **Bing Yao:** Formal analysis (supporting). **Dong Li:** Methodology (supporting); Resources (lead). **Changyan Ma:** Conceptualization (lead); Funding acquisition (lead); Writing‐review & editing (supporting).

## Supporting information

Figure S1

Figure S2

Figure S3

Figure S4

Figure S5

Figure S6

Table S1‐S4

Table S5

Table S6

Supplementary Material

## Data Availability

The data that support the findings of this study are available from the corresponding author upon reasonable request.

## References

[1] Tamma R , Zallone A . Osteoblast and osteoclast crosstalks: from OAF to Ephrin. Inflamm Allergy Drug Targets. 2012;11(3):196‐200. 22280242 10.2174/187152812800392670

[2] Wang Z , Zhang H , Zhang P , Li J , Shan Z , Teng W . Upregulation of miR‐2861 and miR‐451 expression in papillary thyroid carcinoma with lymph node metastasis. Med Oncol. 2013;30(2):577. 23609190 10.1007/s12032-013-0577-9

[3] Golob AL , Laya MB . Osteoporosis: screening, prevention, and management. Med clin North Am. 2015;99(3):587‐606. 25841602 10.1016/j.mcna.2015.01.010

[4] Noma K , Sugiyama T , Cam H , et al. RITS acts in cis to promote RNA interference‐mediated transcriptional and post‐transcriptional silencing. Nat Genet. 2004;36(11):1174‐1180. 15475954 10.1038/ng1452

[5] Landgraf P , Rusu M , Sheridan R , et al. A mammalian microRNA expression atlas based on small RNA library sequencing. Cell. 2007;129(7):1401‐1414. 17604727 10.1016/j.cell.2007.04.040 PMC2681231

[6] Chen CZ , Li L , Lodish HF , Bartel DP . MicroRNAs modulate hematopoietic lineage differentiation. Science. 2004;303(5654):83‐86. 14657504 10.1126/science.1091903

[7] Elbarbary RA , Miyoshi K , Myers JR , et al. Tudor‐SN‐mediated endonucleolytic decay of human cell microRNAs promotes G1/S phase transition. Science. 2017;356(6340):859‐862. 28546213 10.1126/science.aai9372 PMC5551500

[8] Kornfeld JW , Baitzel C , Könner AC , et al. Obesity‐induced overexpression of miR‐802 impairs glucose metabolism through silencing of Hnf1b. Nature. 2013;494(7435):111‐115. 23389544 10.1038/nature11793

[9] Rayner KJ , Suarez Y , Davalos A , et al. MiR‐33 contributes to the regulation of cholesterol homeostasis. Science. 2010;328(5985):1570‐1573. 20466885 10.1126/science.1189862 PMC3114628

[10] Zadran S , Remacle F , Levine RD . miRNA and mRNA cancer signatures determined by analysis of expression levels in large cohorts of patients. Proc Natl Acad Sci USA. 2013;110(47):19160‐19165. 24101511 10.1073/pnas.1316991110 PMC3839764

[11] Wang Y , Wang K , Hu Z , et al. MicroRNA‐139‐3p regulates osteoblast differentiation and apoptosis by targeting ELK1 and interacting with long noncoding RNA ODSM. Cell Death Dis. 2018;9(11):1107. 30382082 10.1038/s41419-018-1153-1 PMC6208413

[12] Chen L , Holmstrom K , Qiu W , et al. MicroRNA‐34a inhibits osteoblast differentiation and in vivo bone formation of human stromal stem cells. Stem Cells. 2014;32(4):902‐912. 24307639 10.1002/stem.1615

[13] Gao Y , Xiao F , Wang C , Cui P , Zhang X , Chen X . Long noncoding RNA MALAT1 promotes osterix expression to regulate osteogenic differentiation by targeting miRNA‐143 in human bone marrow‐derived mesenchymal stem cells. J Cell Biochem. 2018;119(8):6986‐6996. 29741283 10.1002/jcb.26907

[14] Xia P , Gu R , Zhang W , et al. MicroRNA‐200c promotes osteogenic differentiation of human bone mesenchymal stem cells through activating the AKT/beta‐Catenin signaling pathway via downregulating Myd88. J Cell Physiol. 2019;234(12):22675‐22686. 31152447 10.1002/jcp.28834

[15] Li G , An J , Han X , Zhang X , Wang W , Wang S . Hypermethylation of microRNA‐149 activates SDF‐1/CXCR4 to promote osteogenic differentiation of mesenchymal stem cells. J Cell Physiol. 2019;234(12):23485‐23494. 31206187 10.1002/jcp.28917

[16] Ai G , Meng M , Wang L , et al. microRNA‐196a promotes osteogenic differentiation and inhibit adipogenic differentiation of adipose stem cells via regulating beta‐catenin pathway. Am J Transl Res. 2019;11(5):3081‐3091. 31217877 PMC6556631

[17] Lozano C , Duroux‐Richard I , Firat H , Schordan E , Apparailly F . MicroRNAs: key regulators to understand osteoclast differentiation? Front Immunol. 2019;10:375. 30899258 10.3389/fimmu.2019.00375 PMC6416164

[18] Janssen HLA , Reesink HW , Lawitz EJ , et al. Treatment of HCV infection by targeting microRNA. N Engl J Med. 2013;368(18):1685‐1694. 23534542 10.1056/NEJMoa1209026

[19] van Zandwijk N , Pavlakis N , Kao SC , et al. Safety and activity of microRNA‐loaded minicells in patients with recurrent malignant pleural mesothelioma: a first‐in‐man, phase 1, open‐label, dose‐escalation study. Lancet Oncol. 2017;18(10):1386‐1396. 28870611 10.1016/S1470-2045(17)30621-6

[20] Zhang G , Qin L , Hung WY , et al. Flavonoids derived from herbal Epimedium Brevicornum Maxim prevent OVX‐induced osteoporosis in rats independent of its enhancement in intestinal calcium absorption. Bone. 2006;38(6):818‐825. 16413840 10.1016/j.bone.2005.11.019

[21] Xu Y , Li L , Tang Y , Yang J , Jin Y , Ma C . Icariin promotes osteogenic differentiation by suppressing Notch signaling. Eur J Pharmacol. 2019;865:172794. 31733213 10.1016/j.ejphar.2019.172794

[22] Cao H , Ke Y , Zhang Y , Zhang CJ , Qian W , Zhang GL . Icariin stimulates MC3T3‐E1 cell proliferation and differentiation through up‐regulation of bone morphogenetic protein‐2. Int J Mol Med. 2012;29(3):435‐439. 22109711 10.3892/ijmm.2011.845

[23] Wu Y , Xia L , Zhou Y , Xu Y , Jiang X . Icariin induces osteogenic differentiation of bone mesenchymal stem cells in a MAPK‐dependent manner. Cell Prolif. 2015;48(3):375‐384. 25867119 10.1111/cpr.12185 PMC6496185

[24] Wang X , Zhou Z , Zhang T , et al. Overexpression of miR‐664 is associated with poor overall survival and accelerates cell proliferation, migration and invasion in hepatocellular carcinoma. Onco Targets Ther. 2019;12:2373‐2381. 30992673 10.2147/OTT.S188658 PMC6445241

[25] Li X , Zhou C , Zhang C , et al. MicroRNA‐664 functions as an oncogene in cutaneous squamous cell carcinomas (cSCC) via suppressing interferon regulatory factor 2. J Dermatol Sci. 2019;94(3):330‐338. 31138473 10.1016/j.jdermsci.2019.05.004

[26] Wu L , Li Y , Li J , Ma D . MicroRNA‐664 targets insulin receptor substrate 1 to suppress cell proliferation and invasion in breast cancer. Oncol Res. 2019;27(4):459‐467. 29495974 10.3727/096504018X15193500663936 PMC7848467

[27] Shui Y , Yu X , Duan R , et al. miR‐130b‐3p inhibits cell invasion and migration by targeting the Notch ligand Delta‐like 1 in breast carcinoma. Gene. 2017;609:80‐87. 28163094 10.1016/j.gene.2017.01.036

[28] Qu S , Wu J , Bao Q , et al. Osterix promotes the migration and angiogenesis of breast cancer by upregulation of S100A4 expression. J Cell Mol Med. 2019;23(2):1116‐1127. 30450809 10.1111/jcmm.14012 PMC6349213

[29] Karner CM , Lee SY , Long F . Bmp induces osteoblast differentiation through both Smad4 and mTORC1 signaling. Mol Cell Biol. 2017;37(4):e00253‐e316. 10.1128/MCB.00253-16 PMC5288572 27920253

[30] Nakashima K , Zhou X , Kunkel G , et al. The novel zinc finger‐containing transcription factor osterix is required for osteoblast differentiation and bone formation. Cell. 2002;108(1):17‐29. 11792318 10.1016/s0092-8674(01)00622-5

[31] Bellavia D , De Luca A , Carina V , et al. Deregulated miRNAs in bone health: epigenetic roles in osteoporosis. Bone. 2019;122:52‐75. 30772601 10.1016/j.bone.2019.02.013

[32] Wang X , Guo B , Li Q , et al. miR‐214 targets ATF4 to inhibit bone formation. Nat Med. 2013;19(1):93‐100. 23223004 10.1038/nm.3026

[33] Krzeszinski JY , Wei W , Huynh H , et al. miR‐34a blocks osteoporosis and bone metastasis by inhibiting osteoclastogenesis and Tgif2. Nature. 2014;512(7515):431‐435. 25043055 10.1038/nature13375 PMC4149606

[34] Hartke JR . Preclinical development of agents for the treatment of osteoporosis. Toxicol Pathol. 1999;27(1):143‐147. 10367689 10.1177/019262339902700126

[35] ElSharawy A , Keller A , Flachsbart F , et al. Genome‐wide miRNA signatures of human longevity. Aging Cell. 2012;11(4):607‐616. 22533606 10.1111/j.1474-9726.2012.00824.x

[36] Lee BP , Burić I , George‐Pandeth A , et al. MicroRNAs miR‐203‐3p, miR‐664‐3p and miR‐708‐5p are associated with median strain lifespan in mice. Sci Rep. 2017;7:44620. 28304372 10.1038/srep44620 PMC5356331

[37] Wu M , Chen G , Li YP . TGF‐beta and BMP signaling in osteoblast, skeletal development, and bone formation, homeostasis and disease. Bone Res. 2016;4:16009. 27563484 10.1038/boneres.2016.9 PMC4985055

[38] Choi H , Ahn YH , Kim TH , et al. TGF‐beta signaling regulates cementum formation through osterix expression. Sci Rep. 2016;6:26046. 27180803 10.1038/srep26046 PMC4867644

[39] Huang Y , Zheng Y , Jia L , Li W . Long noncoding RNA H19 promotes osteoblast differentiation via TGF‐beta1/Smad3/HDAC signaling pathway by deriving miR‐675. Stem Cells. 2015;33(12):3481‐3492. 26417995 10.1002/stem.2225

[40] Liu K , Jing Y , Zhang W , et al. Silencing miR‐106b accelerates osteogenesis of mesenchymal stem cells and rescues against glucocorticoid‐induced osteoporosis by targeting BMP2. Bone. 2017;97:130‐138. 28108317 10.1016/j.bone.2017.01.014

[41] Bhushan R , Grunhagen J , Becker J , Robinson PN , Ott CE , Knaus P . miR‐181a promotes osteoblastic differentiation through repression of TGF‐beta signaling molecules. Int J Biochem Cell Biol. 2013;45(3):696‐705. 23262291 10.1016/j.biocel.2012.12.008

[42] Arfat Y , Basra MAR , Shahzad M , Majeed K , Mahmood N , Munir H . miR‐208a‐3p suppresses osteoblast differentiation and inhibits bone formation by targeting ACVR1. Mol Ther Nucleic Acids. 2018;11:323‐336. 29858067 10.1016/j.omtn.2017.11.009 PMC5992884

[43] Li H , Fan J , Fan L , et al. MiRNA‐10b reciprocally stimulates osteogenesis and inhibits adipogenesis partly through the TGF‐beta/SMAD2 signaling pathway. Aging Dis. 2018;9(6):1058‐1073. 30574418 10.14336/AD.2018.0214 PMC6284771

