# Supplementary figures and images for: MiR‐664‐3p suppresses osteoblast differentiation and impairs bone formation via targeting Smad4 and Osterix
Source: J Cell Mol Med. 2021 May 4;25(11):5025–37. doi: 10.1111/jcmm.16451 (PMC8178280; doi:10.1111/jcmm.16451)

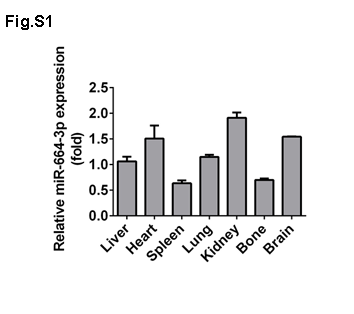

Supplement: Supplementary file 1 — Figure S1 [file JCMM-25-5025-s006.tif]

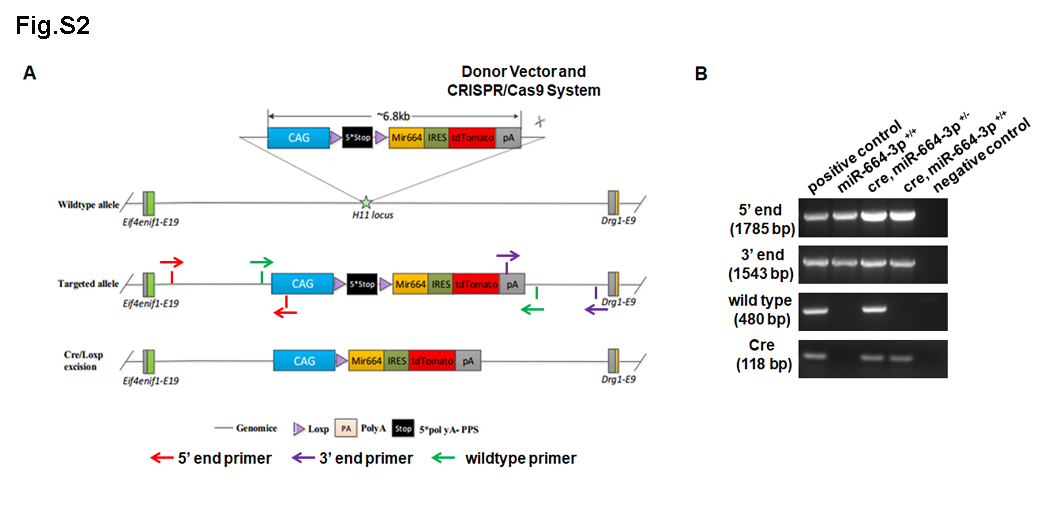

Supplement: Supplementary file 2 — Figure S2 [file JCMM-25-5025-s003.tif]

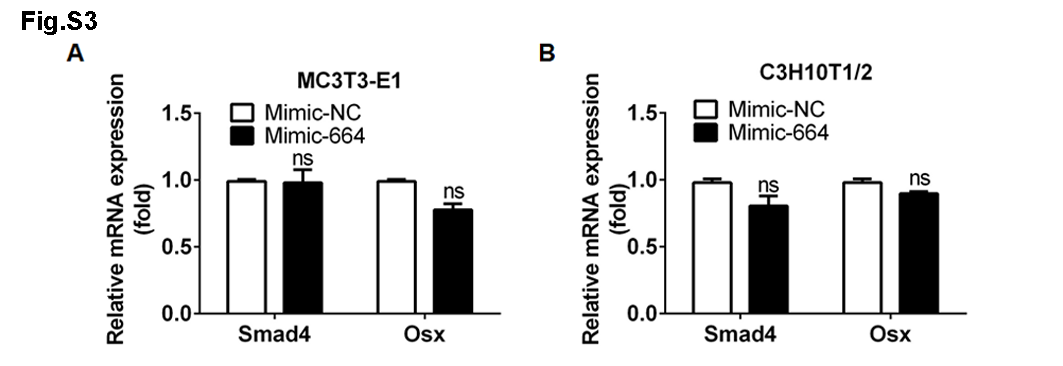

Supplement: Supplementary file 3 — Figure S3 [file JCMM-25-5025-s004.tif]

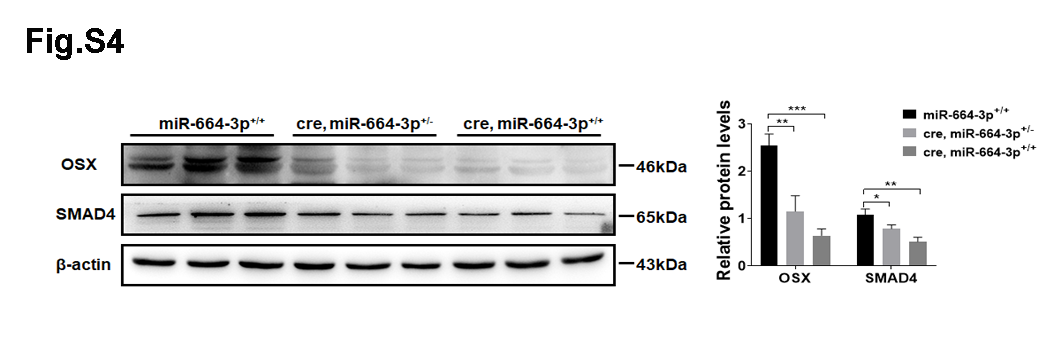

Supplement: Supplementary file 4 — Figure S4 [file JCMM-25-5025-s009.tif]

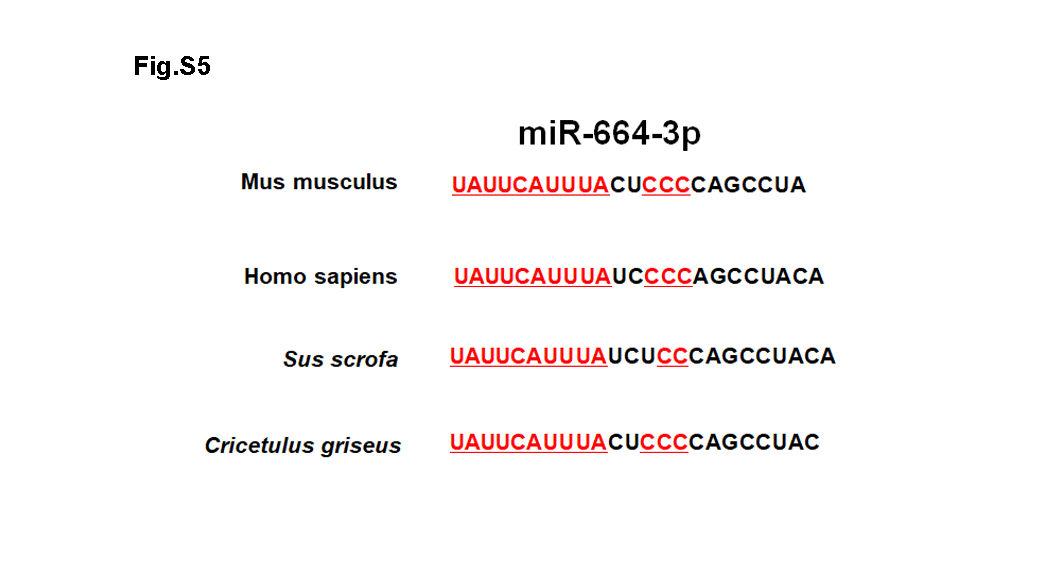

Supplement: Supplementary file 5 — Figure S5 [file JCMM-25-5025-s005.tif]

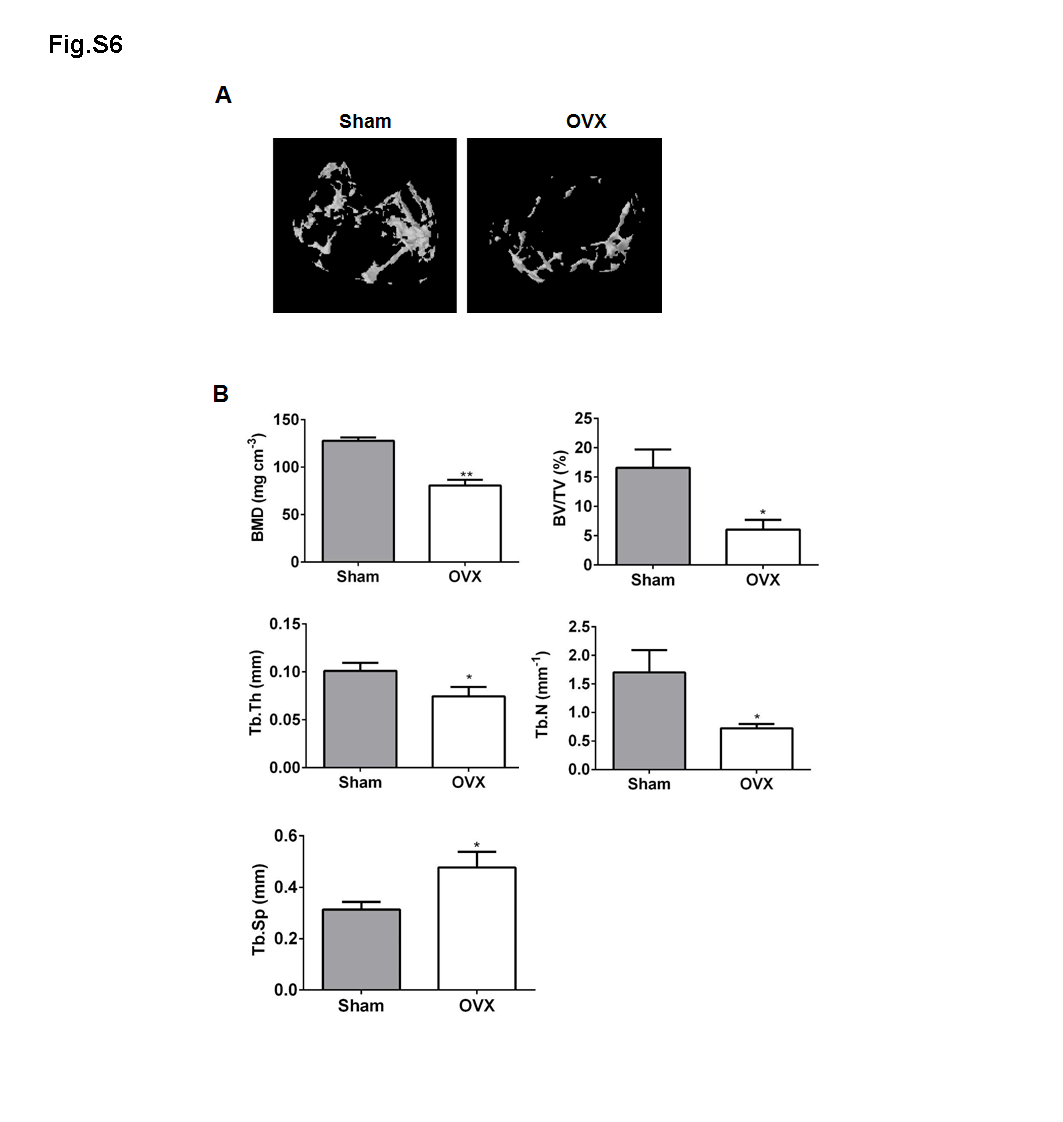

Supplement: Supplementary file 6 — Figure S6 [file JCMM-25-5025-s001.tif]
