# Supplementary material for: MiR‐664‐3p suppresses osteoblast differentiation and impairs bone formation via targeting Smad4 and Osterix
Source: J Cell Mol Med. 2021 May 4;25(11):5025–37. doi: 10.1111/jcmm.16451 (PMC8178280; doi:10.1111/jcmm.16451)
Supplement: Supplementary file 7 — Table S1‐S4 [file JCMM-25-5025-s010.docx]

**Table S1 Primers used for qRT-PCR**

| Name | Primer sequences 5'→3' |
| --- | --- |
| *Runx2*-F  *Runx2*-R | ATGATGACACTGCCACCTCTGAC  AACTGCCTGGGGTCTGAAAAAGG |
| *Osx*-F  *Osx*-R | AGCGACCACTTGAGCAAACAT  GCGGCTGATTGGCTTCTTCT |
| *Alp*-F  *Alp*-R | TGACCTTCTCTCCTCCATCC  CTTCCTGGGAGTCTCATCCT |
| *Ocn*-F  *Ocn*-R | TGCTTGTGACGAGCTATCAG  GAGGACAGGGAGGATCAAGT |
| *Bsp*-F  *Bsp*-R | AAGCAGCACCGTTGAGTATGG  CCTTGTAGTAGCTGTATTCATCCTC |
| *Col1α1*-F  *Col1α1*-R | GCAACAGTCGCTTCACCTACA  CAATGTCCAAGGGAGCCACAT |
| *Smad4*-F  *Smad4*-R  *OSX*-F  *OSX*-R  *SMAD4*-F  *SMAD4*-R  *β-actin*-F  *β-actin*-R | AGCCGTCCTTACCCACTGAA  GGTGGTAGTGCTGTTATGATGGT  CTCCTTTCACCTGCAGGCAG  CAGACAGTCAGAAGAGCTGT  AGGACAGAAGCCATTGAG  CGATGACACTGACGCAAA  AGATGTGGATCAGCAAGCAG  GCGCAAGTTAGGTTTTGTCA |

**Table S2 Primers used for stem-loop qRT-PCR**

| Name | Primer sequences 5'→3' |
| --- | --- |
| mmu-miR-664-3p | **RT:**GTCGTATCCAGTGCAGGGTCCGAGGTATTCGCACTGGATACGACTAGGCT |
|  | **Forward:** AGCCCGTATTCATTTACTCCCC |
| hsa-miR-664-3p | **RT:**GTCGTATCCAGTGCAGGGTCCGAGGTATTCGCACTGGATACGACTGTAGGCT |
|  | **Forward:** AGCCCGTATTCATTTATCCCC |
| mmu-miR-27a-3p | **RT:** GTCGTATCCAGTGCAGGGTCCGAGGTATTCG  CACTGGATACGACGCGGAACT |
|  | **Forward:** GCGGGCGTTCACAGTGGCTA |
| mmu-miR-32-3p | **RT:** GTCGTATCCAGTGCAGGGTCCGAGGTATTCG  CACTGGATACGACAATATC |
|  | **Forward:** AGCCCGCAATTTAGTGTGTGT |
| mmu-miR-19b-1-5p | **RT:** GTCGTATCCAGTGCAGGGTCCGAGGTATTCG  CACTGGATACGACGCTGGATG |
|  | **Forward:** AGCCCGAGTTTTGCAGGTTTG |
| mmu-miR-130b-3p | **RT:** GTCGTATCCAGTGCAGGGTCCGAGGTATTCG  CACTGGATACGACATGCCC |
|  | **Forward:** AGCCGCAGTGCAATGATGAAA |
| mmu-miR-17-3p | **RT:** GTCGTATCCAGTGCAGGGTCCGAGGTATTCG  CACTGGATACGACCTACAAGT |
|  | **Forward:** ACGGGCACTGCAGTGAGGGC |
| mmu-miR-29a-5p | **RT:** GTCGTATCCAGTGCAGGGTCCGAGGTATTCG  CACTGGATACGACCTGAAC |
|  | **Forward:** AGCCCGACTGATTTCTTTTGGT |
| mmu-miR-20a-5p | **RT:** GTCGTATCCAGTGCAGGGTCCGAGGTATTCG  CACTGGATACGACCTACCT |
|  | **Forward:** AGCCCGTAAAGTGCTTATAGTGC |
| mmu-miR-204-5p | **RT:** GTCGTATCCAGTGCAGGGTCCGAGGTATTCG  CACTGGATACGACAGGCAT |
|  | **Forward:** AGCCCGTTCCCTTTGTCATCCT |
| mmu-let-7f-5p | **RT:** GTCGTATCCAGTGCAGGGTCCGAGGTATTCG  CACTGGATACGACAACTAT |
|  | **Forward:** GCGGGCTGAGGTAGTAGATTGT |
| mmu-miR-224-5p | **RT:** GTCGTATCCAGTGCAGGGTCCGAGGTATTCG  CACTGGATACGACAACGGA |
|  | **Forward:** GCGGGCGTAAGTCACTAGTGGT |
| mmu-miR-374-5p | **RT:** GTCGTATCCAGTGCAGGGTCCGAGGTATTCG  CACTGGATACGACCACTTA |
|  | **Forward:** AGCCCGGATATAATACAACCTGC |
| mmu-miR-467a-5p | **RT:**GTCGTATCCAGTGCAGGGTCCGAGGTATTCGCACTGGATACGACCGCATA |
|  | **Forward:** AGCCCGTAAGTGCCTGCATGTA |
| mmu-miR-3109-5p | **RT:**GTCGTATCCAGTGCAGGGTCCGAGGTATTCGCACTGGATACGACAGCATGGG |
|  | **Forward:** AGACGAATGGATGCGATGGTT |
| mmu-miR-669f-3p | **RT:**GTCGTATCCAGTGCAGGGTCCGAGGTATTCGCACTGGATACGACATACGT |
|  | **Forward:** AGCCCGCATATACATACACACAC |
| mmu-miR-652-5p | **RT:**GTCGTATCCAGTGCAGGGTCCGAGGTATTCGCACTGGATACGACGAATGG |
|  | **Forward:** GTACACCAACCCTAGGAGGGGGAC |
| mmu-miR-335-3p | **RT:**GTCGTATCCAGTGCAGGGTCCGAGGTATTCGCACTGGATACGACGGTCAG |
|  | **Forward:** GCGGGCTTTTTCATTATTGCTC |
| mmu-miR-26a-5p | **RT:**GTCGTATCCAGTGCAGGGTCCGAGGTATTCGCACTGGATACGACAGCCTA |
|  | **Forward:** AGCCCGTTCAAGTAATCCAGGA |
| mmu-miR-126-5p | **RT:**GTCGTATCCAGTGCAGGGTCCGAGGTATTCGCACTGGATACGACCGCGTA |
|  | **Forward:** AGCCCGCATTATTACTTTTGG |
| mmu-miR-452-5p | **RT:**GTCGTATCCAGTGCAGGGTCCGAGGTATTCGCACTGGATACGACGTCTCA |
|  | **Forward:** AGCCCGTGTTTGCAGAGGAAAC |
| Reverse primer | CAGTGCAGGGTCCGAGGT |

**Table S3 Primers used for plasmid construction**

| Name | Primer sequences 5'→3' |
| --- | --- |
| Smad4-3'UTR-F  Smad4-3'UTR-R | CTCTAGACGCCCTAACCATTTCC  CTCTAGACTCAGTTTTATTTACGCTA |
| Smad4-3'UTR-D1-F  Smad4-3'UTR-D1-R | TGTTAAACAGTATCTTCTAT  ATAGAAGATACTGTTTAACA |
| Smad4-3'UTR-D2-F  Smad4-3'UTR-D2-R | GTACTTCAACTGAAATATTTTGG  CCAAAATATTTCAGTTGAAGTAC |
| Osx-CDS-F  Osx-CDS-R | CTCTAGACCCTCTGCGGGACTCAACAA  CTCTAGAGGGTGCAGGCGAAGTGGAAG |
| Osx-CDS-mut-F  Osx-CDS-mut-R | GGCACAGGATTCAACGGTCGATAT  ATATCGACCGTTGAATCCTGTGCC |
| Osx-CDS-full-F | CGGAATTCATGGCGTCCTCTCTGCTTGA |
| Osx-CDS-full-R | CCCAAGCTTTCAGATCTCTAGCAGGTTGC |
| SMAD4-3'UTR-WT1-F  SMAD4-3'UTR-WT1-R | CTCTAGATGCCATGTGGGTGAGTT  CTCTAGAGGCTGTTGCCTGTCATT |
| SMAD4-3'UTR-Mut1-F  SMAD4-3'UTR-Mut1-R | AACTAAATACTAGCTAAACTGAATA  TATTCAGTTTAGCTAGTATTTAGTT |
| SMAD4-3'UTR-WT2-F  SMAD4-3'UTR-WT2-R | CTCTAGAGGGTTCAACTGATTCTCC  CTCTAGAGCACTGTTCACAGGAGG |
| SMAD4-3'UTR-Mut2-F  SMAD4-3'UTR-Mut2-R | GTCATCTTTGAGACCTAAGACTAAA  TTTAGTCTTAGGTCTCAAAGATGAC |
| SMAD4-3'UTR-WT3-F  SMAD4-3'UTR-WT3-R | CTCTAGAACTTGAATGCTGCTCTT  CTCTAGAATCTACTCCAACTCCCTC |
| SMAD4-3'UTR-Mut3-F  SMAD4-3'UTR-Mut3-R | AGACTGGAGTAACTACGTGAAACTT  AAGTTTCACGTAGTTACTCCAGTCT |
| OSX-3'UTR-WT-F  OSX-3'UTR-WT-R | CTCTAGAGCTCCCAACACTATTTCTC  CTCTAGACCACCCATTCTTCAGG |
| OSX-3'UTR-Mut-F  OSX-3'UTR-Mut-R  pcDNA-Osx-CDS-F  pcDNA-Osx-CDS-R  pcDNA- Smad4-3'UTR-F  pcDNA- Smad4-3'UTR-R | CCAGGAGTAACTGCTAGTAATAATT  AATTATTACTAGCAGTTACTCCTGG  CGAAGCTTCGCCCTAACCATTTCC  CCCGAATTCCTCAGTTTTATTTACGCTA  CGAAGCTTCCCTCTGCGGGACTCAACAA  CCCGAATTCGGGTGCAGGCGAAGTGGAAG |

**Table S4 Primers used for genotyping**

| Name | Primer sequences 5'→3' |
| --- | --- |
| Cre-F | ACAATCAAGGGTCCCCAAAC |
| Cre-R | CCAGCCGCAAAGAGTCTACA |
| 5’end-F | CATGCCCACCAAAGTCATCAG |
| 5’end-R | TGTACTGGGCATAATGCCAGG |
| 3’end-F | CCTTGGCCTCCCAAATTGCTG |
| 3’end-R | CTCCTGAGGTCTAAGGGAACCTCG |
| wildtype-F | TGTGGATCAGGGCAGTCTGGT |
| wildtype-R | CTTGTGGGTCTTCCACCTTTCTTC |
