# Supplementary material for: MiR‐664‐3p suppresses osteoblast differentiation and impairs bone formation via targeting Smad4 and Osterix
Source: J Cell Mol Med. 2021 May 4;25(11):5025–37. doi: 10.1111/jcmm.16451 (PMC8178280; doi:10.1111/jcmm.16451)
Supplement: Supplementary file 10 — Supplementary Material [file JCMM-25-5025-s007.docx]

**Supplemental figure legends:**

**Figure S1** qRT-PCR analysis of miR-664-3p levels in different tissues of mice. Data are mean ± SD, n = 3.

# Figure S2 Generation of the conditional miR-664-3p transgenic mice. (A) Schematic representation of the generation of osteoblastic miR-664-3p transgenic mice using CRISPR/Cas9 technology. Top: wild-type allele, middle: targeted allele, bottom: Cre/Loxp excision.  (B) PCR Genotyping of osteoblastic miR-664-3p transgenic mice. miR-664-3p^+/+^ mice showed two bands: 1785 and 1543 bp; cre, miR-664-3p^+/–^ mice showed four bands: 1785, 1543, 480, and 118 bp; cre, miR-664-3p^+/+^ mice showed three bands: 1785, 1543, and 118 bp.

**Figure S3** qRT-PCR analysis of *Smad4* and *Osx* mRNA levels. *Smad4* and *Osx* mRNA levels in MC3T3-E1 (A) and C3H10T1/2 (B) cells after treatment with Mimic-664 or Mimic-NC. Data are mean ± SD, n = 3. ns, no significant difference.

**Figure S4** Western blotting analysis of OSX and SMAD4 protein expression in the bone tissues collected from TG664 mouse lines (cre, miR-664-3p^+/–^ and cre, miR-664-3p^+/+^) and control mice (miR-664-3p^+/+^) at 9 weeks of age. Representative images (left panel) and the densitometric analysis (right panel) are shown. ^*^P < 0.05, and ^**^P < 0.01. ^***^P < 0.001.

**Figure S5** Sequence comparison of mature miR-664-3p among different species.

**Figure S6** Characterization of bone phenotypes in ovariectomized mice. (A) Representative μCT reconstructive images of the femoral metaphysis collected from mice after sham or OVX surgery. (B) Bone morphometric analysis of trabecular bone of the distal femurs isolated from each group. BMD, bone mineral density; BV/TV, bone volume/tissue volume; Tb.Th, trabecular thickness; Tb.N, trabecular number; Tb.Sp, trabecular separation. Data are mean ± SD, *n* = 5 mice in each group. ^*^P < 0.05, and ^**^P < 0.01.
